# Comparison of an Enzyme Linked-Immunosorbent Assay and a Chemiluminescent Immunoassay with an Immunofluorescence Assay for Detection of Phase II IgM and IgG Antibodies to *Coxiella burnetii*

**DOI:** 10.3390/microorganisms12030552

**Published:** 2024-03-11

**Authors:** Juan Francisco Gutiérrez-Bautista, María Tarriño, Adrián González, María José Olivares Durán, Fernando Cobo, Juan Antonio Reguera, Javier Rodríguez-Granger, Antonio Sampedro

**Affiliations:** 1 Departamento de Bioquímica, Biología Molecular e Inmunología III, University of Granada, 18016 Granada, Spain; juanf.gutierrez.bautista.sspa@juntadeandalucia.es; 2 Servicio de Análisis Clínicos e Inmunología, University Hospital Virgen de las Nieves, 18014 Granada, Spain; mjolivaresduran@gmail.com; 3 Instituto de Investigación Biosanitaria de Granada (ibs.GRANADA), 18012 Granada, Spain; jantonio.reguera.sspa@juntadeandalucia.es (J.A.R.); javierm.rodriguez.sspa@juntadeandalucia.es (J.R.-G.); antonio.sampedro.sspa@juntadeandalucia.es (A.S.); 4 Servicio de Microbiología, University Hospital Virgen de las Nieves, 18014 Granada, Spain; maria.tarrino.sspa@juntadeandalucia.es (M.T.); adriangonzalezmartinez1@gmail.com (A.G.)

**Keywords:** *Coxiella burnetii*, Q fever, diagnosis, enzyme-linked immunoassay, chemiluminescent immunoassay

## Abstract

In this study, we have compared the detection of IgM and IgG against *C. burnetii* phase II of an enzyme-linked immunosorbent assay (ELISA) (Euroimmun) and a chemiluminescent immunoassay (CLIA) (VIRCLIA, Vircell). In addition, an indirect immunofluorescence assay (IFA) was used as a reference test. One hundred forty-eight sera were used for IgG evaluation, and eighty-eight for IgM. The sensitivity of ELISA and CLIA in detecting phase II IgM was excellent. On the other hand, the CLIA IgM showed better specificity than the ELISA IgM. As for phase II IgG, the specificity of ELISA and CLIA was similar, while the ELISA technique showed a higher sensitivity. In conclusion, the best system to detect phase II IgM antibodies against *C. burnetii* is the CLIA from Vircell, which is characterized by high sensitivity and specificity. For the detection of phase II IgG, the Euroimmun ELISA and Vircell CLIA assays are suitable for the determination of this marker in the laboratory, although the IgG ELISA has greater sensitivity.

## 1. Introduction

Q fever is a zoonotic disease caused by the intracellular bacterium *Coxiella burnetii*. Many animals serve as reservoirs of infection, including domestic mammals, marine mammals, reptiles, ticks, and birds [1], but these are mostly of little significance for humans. Some domestic ruminants are the main source of infection for humans. In females, after an initial infection, *C. burnetii* multiplies in the placenta and is eliminated in large quantities in the products of conception. Transmission to humans occurs through the inhalation of aerosolized bacteria released into the environment after delivery or abortion. These aerosols may also be created long after diseased animals have released germs into the environment due to *C. burnetii*’s ability to survive for extended durations in soil. Other routes of human infection, such as tickborne and digestive, could also be involved but would be less effective [2]. Q fever is a zoonosis; therefore, interhuman transmission has been described anecdotally [3].

The disease has a wide geographical distribution, and because *C. burnetii* is commonly found in livestock, Q fever is frequently associated with occupational exposure, particularly in professions that involve contact with livestock, such as veterinarians or farmers who have a higher risk of contracting the disease. The number of notifications per 100,000 inhabitants in the EU/EEA was 0.2 for 2019. Spain has the highest case notification (0.7 cases per 100,000), followed by Romania (0.6 cases per 100,000) and Bulgaria (0.5 cases per 100,000) [4]. In Spain, most of the confirmed cases were documented in the northern regions, specifically in the provinces of Basque and Navarre, where there is greater livestock-related activity [5].

Q fever infection can present as either acute (primary) or chronic (localized) infections. Although up to 50% of initial infections are believed to be asymptomatic, noticeable symptoms in cases of acute infection typically appear as a non-specific febrile illness after a 2 to 3-week incubation period. Other symptoms of acute infections include a severe retro-orbital headache, fever, chills, malaise, and myalgia [6]. In some instances, acute Q fever may progress to atypical pneumonia, ranging from mild to severe [7]. Another common presentation is isolated hepatitis, characterized by fever, abdominal pain, nausea, and occasionally vomiting, with elevated hepatic enzymes [2]. A small percentage of primary Q fever infections will enter a latent phase and reappear as a more severe and focalized infection or chronic Q fever. Q fever endocarditis is the most frequently reported form of persistent *C. burnetii* infection being associated with underlying heart valve disease [8]. Other types of chronic Q fever infections are vascular infections, osteoarticular infections, and more exceptionally persistent lymphadenitis [2].

The expression of *C. burnetii* surface lipopolysaccharides is subject to phase variation; when the bacterium is isolated from infected animals, it is expressed in the phase I form, while passage through cell culture or embryonated eggs produces phase II antigen expression [9]. This phase is much less virulent than phase I. The immune response induces the production of anti-phase II and anti-phase I antibodies. Phase II IgM antibodies appear first, 7 to 10 days from the onset of symptoms, followed by the appearance of IgG 5 to 6 days later [2]. The decrease in levels of phase II IgG occurs slowly, becoming detectable at 6 months, and may remain at low titers for years or even a lifetime. Phase II IgM is still detectable in a major part of the samples at 12 months [10]. Antibodies against phase I appear later, and high phase I IgG titers are associated with persistent fever. A high titer of phase I antibodies, greater than or equal to the phase II antibody titer, correlates with a higher probability of *C. burnetii* endocarditis [1]. For that reason, an investigation for persistent infection should be performed in the case of sustained high levels of phase I antibodies 6 months after completion of treatment [2].

Diagnosing acute infection includes direct and indirect methods. Direct techniques for diagnosing Q fever are most effective when applied to patients who show clinical symptoms of acute Q fever within 14 days of the onset of symptoms or to those who have chronic Q fever [11]. As for the culture, *C. burnetii* can be isolated in conventional cell cultures in a wide variety of cell lines, including all fibroblast cell lines. After an incubation period of 5 to 15 days, *C. burnetii*-infected cells are detected as cytoplasmic inclusions. Due to the extreme infectivity of this organism (Biosafety Level 3 [BSL-3]), culture is not used for routine diagnosis. Currently, detection of *C. burnetii* DNA by PCR in various clinical samples is available for diagnosis. In early acute forms, the microorganism can be detected in blood up to 15 days after the onset of symptoms when specific IgG antibodies appear [12]. Since most samples are sent to the laboratory with an undetermined duration of evolution or after 2 weeks from the onset of symptoms, diagnosis is mainly based on serologic tests for the detection of specific antibodies against phase I and II antigens. Demonstration of seroconversion or a four-fold increase in IgG antibody titers against the phase II antigen by an IFA test between samples collected during the acute and convalescent stages of infection in a patient with symptoms confirms the diagnosis. Serum samples taken during the acute phase should be gathered within 7–10 days from the start of symptoms, while the convalescent sample should be collected 3–6 weeks thereafter [11]. If paired sera are not available, a single sample with a phase II IgG titer ≥ 1:128 may indicate acute Q fever. However, a sample collected during the acute stage of the disease, very early, can lead to a false negative result if the test is performed in the absence of a convalescent sample because the antibodies usually do not develop until more than 7 days post-symptom onset [11]. IgM phase II is the first class of antibodies to be detected in blood, followed by IgG phase II [2]. Determination of phase II IgM antibodies can be used as a screening test for presumptive diagnosis of acute Q fever. The serological diagnosis of acute Q fever based on a single serum sample by determination of phase II IgM may be imprecise since phase II IgM can persist in serum for long periods of time [13]. Furthermore, an isolated IgM result may be a false positive result due to cross-reactivity with other infectious agents [14,15]. 

The most common serological methods for testing antibodies against *C. burnetii* are complement fixation (CF), enzyme-linked immunosorbent assay (ELISA), and indirect immunofluorescent antibody (IFA) tests. Other techniques, such as Western blotting, dot immunoblotting, radioimmunoassay, microagglutination, and the indirect hemolysis test, exist but are much less common. Historically, the most commonly used technique has been CF, but while this method exhibits good specificity, its sensitivity is low. A comparative study between CF and IFA revealed that the CF assay failed to detect IgM antibodies in any of the samples analyzed during the first week from the onset of symptoms, whereas IFA was able to detect them 3 days after symptom onset. This lower sensitivity of CF was evident even 3 weeks after the onset of symptoms [16]. IFA is the assay considered as the reference in the serological diagnosis of Q fever and is the method mentioned by the Centers for Disease Control and Prevention (CDC) for defining confirmed cases in acute and chronic *C. burnetii* infection [11]. The drawbacks of this test are attributed to the subjective nature of visualization, leading to potential variations between laboratories. Additionally, variations in antigen preparations and assay protocols can impact the final result titers [11]. ELISA methods are recognized by the CDC for use in the diagnosis of acute and chronic *C. burnetii* infection. The assay offers advantages such as ease of execution, objective interpretation, and the potential for automation, making it suitable for screening when dealing with a high number of samples and for use in epidemiological studies.

There are numerous assays on the market in chemiluminescent format for serological diagnosis of infectious diseases, and their validity has been verified. Recently, a new automated chemiluminescent immunoassay (CLIA) was developed by Vircell (Granada, Spain) for Q fever serological diagnosis, but there are no publications that support the performance of this new test. Laboratories contemplating the use of such assays must thoroughly validate their performance characteristics against accepted reference methods before introducing them into the clinical setting.

The purpose of this study has been to compare an ELISA and a new CLIA in the detection of phase II IgG and IgM and to evaluate their sensitivity and specificity using a commercial IFA assay as a reference method.

## 2. Materials and Methods

### 2.1. Samples

A total of 148 stored (−20 °C) sera were used for this study. All samples were from patients with clinical suspicion of acute infection and sent to our laboratory for diagnosis of Q fever. Two panels of sera with known phase II IgM and IgG results by IFA were utilized: Panel I consisted of 88 sera with a positive IFA result for phase II IgG. Among these sera, 26 also tested positive for IgM, and 62 had a negative result for IgM. Panel II included 60 sera with negative IFA results for phase II IgG. For the assessment of CLIA IgM and ELISA Euroimmun IgM, only Panel I was used. All sera, both from Panel I and Panel II, were employed for the evaluation of the two IgG assays.

### 2.2. Serological Test

The sera were thawed at room temperature and assayed by CLIA kit (*C. burnetii* VIRCLIA^®^ IgM Monotest and IgG Monotest, Vircell, Granada, Spain) and ELISA kit (*C. burnetii* phase 2 IgM/IgG, Euroimmun, Lübeck, Germany). The assays were performed according to the manufacturer’s instructions. The results of the samples by IFA were previously known, but for the purpose of this study, the samples with discrepant results were retested by IFA.

### 2.3. Commercial Indirect Immunofluorescence Assay

A commercial IFA kit (*Coxiella burnetii* I + II IFA IgM/IgG/IgA, Vircell, Spain). The kit contains slides with pairs of wells where the *C. burnetii* Nine Mile strain is placed. Each pair contains one well with phase I antigen and another with phase II antigen. In our center, a serological diagnosis of Q fever is carried out using this test. This assay has been considered the gold standard to compare ELISA and CLIA assays. IFA assay was carried out following the manufacturer’s protocols with the exception of the first dilution step: serum samples were diluted four-fold, and a titer ≥ 1:25, as the first dilution point, was considered positive for IgM and a titer of 1:100 for IgG. For the detection of IgM, the samples are previously diluted (1:5) in an IFA sorbent solution, which avoids possible interference due to the presence of IgG. Dilutions of the pretreated or direct sample were carried out in phosphate buffer saline (PBS) pH 7.2. Then, 25 µL of the appropriate dilution of each sample was deposited onto slides containing the antigens, and the slides were incubated (37 °C for 30 min), washed with PBS, and then dried in a humidity chamber. Then, 25 µL of fluorescein isothiocyanate (FITC)-anti-IgM or anti-IgG antibody was added to the concavities. The slides were incubated (37 °C for 30 min), washed, and read using a fluorescence microscope with 400× magnification.

### 2.4. Commercial Chemiluminiscent Immunoassays (CLIA) 

The CLIA manufactured by Vircell is an indirect immunoassay that uses chemiluminescence as a detection method. The test is presented in monotest format (microwell strip) and is ready to use. Sample processing, including predilution and/or treatment, and analysis are carried out automatically on the Lotus instrument without the need for pipetting by the operator. The Lotus platform allows continuous loading of samples (primary tube) and monotest strips. Each monotest strip is composed of 3 reaction wells and 5 reagent wells. In the reaction wells coated with antigen, the calibrator, sample, and negative control are processed simultaneously. Reagent wells contain ready-to-use all necessary reagents (diluent, conjugate, chemiluminescent substrate), including a negative control and a calibrator for each sample. The calibrator, set in a range, enables the interpretation of the sample and acts as an intra-assay positive control monitoring that the washing is correct, there is no over-incubation, and that the reagents have performed correctly. Microwell strips were incubated with clinical specimens (calibrator and negative control), and after the washing step, the plate was incubated with a conjugate reagent. Then, after the second washing step, the chemiluminescent substrate was added, followed by further incubation. The product resulting from the action of the conjugate on the substrate emits light (Relative Light Units or RLU) that is measured. The calibrator control included in each assay is used to calculate an index for each sample (Index = Relative Light Units (RLU) Sample/Relative Light Units (RLU) calibrator). Samples with Index > 1.1 were considered positive, and those <0.9 were taken as negative. Samples with an Index between 0.9 and 1.1 are considered equivocal or borderline.

### 2.5. Enzyme-Linked Immunosorbent Assay (ELISA)

EUROIMMUN Phase II IgM/IgG ELISA uses wells coated with phase II *C. burnetii* antigens. Serum samples were diluted 1:100 in sample buffer and transferred (100 µL) to the well. The plates were incubated for one hour at 37 °C, followed by three washes with wash buffer. Anti-human IgM or anti-human IgG conjugate (100 μL) was added and incubated for 30 min at 37 °C. After three washes, 100 µL substrate solution was dispensed and incubated for 15 min at room temperature in the dark. Stop solution (100 μL) was added into each of the microplate wells, and the optical density was read at a wavelength of 450 nm. The results can be evaluated by calculating a ratio of the extinction value of the patient sample over the extinction value of the calibrator. The samples with ratio values ≥ 1.1 were considered positive, and those ≤0.8 were taken as negative. Samples with ratios between 0.8 and 1.1 are considered doubtful. The ELISA assay was processed on a fully automated ELISA processing system (Euroimmun Analyzer I, Euroimmun, Lübeck, Germany).

### 2.6. Statistical Analysis

Sensitivity and specificity values were calculated with their 95% confidence intervals, and for these estimates, indeterminate results in the ELISA or CLIA were considered the most adverse. The z-test is used to compare two proportions to determine the statistical significance between the parameters of the two assays. Values with *p* < 0.05 were considered statistically significant. The Kappa coefficient was used to measure agreement between the assays to be evaluated and the reference test. A Kappa coefficient > 0.79 was considered excellent agreement; values of 0.6 to 0.79 represent good, and values of 0.4 to 0.59 represent moderate agreement [17].

## 3. Results

### 3.1. Detection of Phase II IgG

A concordant result was obtained in 121 (82%) of the 148 sera tested. The agreement between CLIA and ELISA in IgG detection was good (K = 0.65, CI 95%: 0.82–0.48). Of the 148 sera tested for IgG, 85 (57%) were positive with ELISA, and 77 (52%) were positive with CLIA. Of the 88 positive samples with IFA, 68 were concordant in both ELISA and CLIA assays, and 20 were discordant. The discordant results in the panel I consisted of 10 sera positive by ELISA and negative by CLIA, four samples positive by ELISA and equivocal by CLIA, two sera equivocal with ELISA and negative with CLIA, three sera equivocal with ELISA and positive with CLIA, and one sample negative by ELISA but equivocal with CLIA. In panel II, of the 60 negative samples with IFA, 53 sera were concordant (52 samples with negative and one sample with positive results by both ELISA and CLIA kits), seven were discordant (two sera positive by ELISA and negative by CLIA, and five sera positive with CLIA and negative with ELISA) (Table 1).

### 3.2. Detection of Phase II IgM

Concordant results were obtained for 66 (75%) of the 88 sera; agreement between CLIA and ELISA in IgM detection was moderate (K = 0.5, CI 95%: 0.69–0.28). Of the 26 positive samples with IFA, 23 were concordant by ELISA and CLIA, and three samples were discordant (two sera with a positive result with ELISA and negative by CLIA and one serum negative by ELISA and positive by CLIA). 

Of the 62 negative samples with IFA, 43 were concordant in ELISA and CLIA, three samples with negative results with IFA were positive by both ELISA and CLIA, and 19 were discordant (11 sera positive by ELISA and negative by VICLIA, four sera with a borderline result by ELISA and negative by CLIA, three samples negative with ELISA and positive with CLIA and one serum positive by ELISA and borderline by CLIA) (Table 2).

### 3.3. Sensitivity and Specificity of ELISA and CLIA

The results of sensitivity and specificity in the detection of IgM and IgG in both assays are shown in Table 3. In this table, the equivocal results obtained by ELISA or CLIA have been considered the most adverse.

Regarding the detection of phase II IgG, the specificity of both assays has been higher than 90%, being higher in the ELISA assay than in CLIA (95% and 90%, respectively), although this difference has not been statistically significant (*p* = 0.051). Regarding false negatives, the ELISA technique showed a sensitivity of 93%, compared to 81% for CLIA (*p* = 0.002). 

The sensitivity of the CLIA IgM assay was 92%, while that of the Euroimmun ELISA IgM was 96%, with these differences not being statistically significant (*p* = 0.13). The CLIA IgM assay showed better specificity than the ELISA IgM assay (89% and 69%, respectively). This difference in the specificity of both assays in the detection of IgM has been statistically significant (*p* = 0.001). False-positive cases included four equivocal and 15 negative IgM results for ELISA and one equivocal and six positives for CLIA.

We found excellent or good agreement between the detection of phase II IgG by CLIA or ELISA and the detection of phase II IgG by IFA test (K > 0.6; Table 3). Regarding the agreement in the detection of IgM, the CLIA assay showed good agreement with the IFA assay (K = 0.77), but agreement between ELISA and IFA was only moderate (K = 0.54) (Table 3).

## 4. Discussion

The laboratory diagnosis of acute Q fever is based on serology and PCR in the early stages. Good sensitivity and specificity values in serological assays help establish the diagnosis and subsequent treatment, which will lead to a better result. In the present study, an ELISA and a new CLIA for the detection of phase II IgM and IgG antibodies to *C. burnetii* have been compared and evaluated using the IFA test as a reference. As mentioned above, the IFA test is considered the gold standard in the diagnosis of Q fever [11]; however, it presents difficulties due to its laboriousness and subjective reading. Healy et al. compared the serological results of Q fever from different reference centers in the United Kingdom, France, and Australia. They found that the agreement among the three centers in the interpretation of microimmunofluorescence was only 35% [18].

ELISA assays are accepted for the serological diagnosis of Q fever [11] and have the benefit of being easily executed, with less subjectivity in interpretation compared to the IFA test. Additionally, automation is feasible, which allows for the processing of a high number of samples or as a screening test in the context of outbreaks caused by this agent. The VirClia^®^ system shares objective reading and automation with the ELISA assay and also allows the study of samples in a unitary manner. It is not necessary to accumulate samples to perform the test, and it guarantees the processing of the sample the moment that it is received, thus reducing response time. Another advantage of the VirClia system derives from including the controls on the single-dose strip, so the expense of control strips is unnecessary and the time required to obtain results is less than one hour.

The implementation of a new assay in the laboratory requires, as a preliminary step, its validation against a reference method. To detect phase II-specific IgG and IgM, different ELISAs have been evaluated, especially for the detection of IgM, showing a great variability of results [13,19,20]. However, there are no studies on the validity of *C. burnetii* CLIA.

Determination of phase II IgG by immunoenzymatic assays has a more limited application in serological diagnosis. In a patient with clinical evidence of infection detection of specific IgG together with IgM, it is indicative of acute infection [2], but residual IgG antibody may be detected for years, and its detection in isolation may be due to ongoing or past infection [2]. In the present study, the sensitivity for IgG phase II antibody detection by Euroimmun ELISA kits was 93%, higher than that reporter for other ELISA assays such as Virion/Serion, Mikrogen, IBL, and Biomed kits, which showed sensitivities of 68, 55, 68, 76, and 55%, respectively [19]. The specificity for Euroimmun ELISA IgG was 95%, lower than the 100% reported for Virion/Serion, Mikrogen, and IBL ELISAs [19]. The CLIA IgG assay presents lower sensitivity (81%) and lower specificity (90%) than the Euroimmun ELISA IgG assay. Vircell’s CLIA IgG does have a higher sensitivity than other ELISAs on the market, such as Virion/Serion, Mikrogen, IBL, and Biomed, with similar specificities [19].

However, the detection of phase II IgG antibodies plays a greater role in epidemiological studies than in diagnosis. For the serological diagnosis of acute Q fever, IgM is of major relevance. In this study, the Euroimmun ELISA IgM assay achieved excellent sensitivity (96%) compared to the commercial Nova Lisa IgM, IBL International ELISA IgM, and Biomed ELISA IgM assays [19]. With sensitivity values similar and even higher than those obtained in our study, they have been reported for PanBio [20] and Serion ELISA IgM assays [19]. We obtained a lower sensitivity with the CLIA IgM assay (92%), although the difference with the Euroimmun ELISA IgM was not statistically significant (*p* = 0.13). 

The specificity obtained for the Euroimmun ELISA IgM assay has been moderate (69%) but higher than that reported for other commercial ELISAs such as Serion ELISA IgM, Nova Lisa IgM, IBL International ELISA IgM, and Biomed ELISA IgM assays [19]. The specificity achieved by the CLIA IgM assay has been commendable at 89%, surpassing that of the Euroimmun ELISA IgM. This disparity in specificity has been determined to be statistically significant (*p* = 0.002).

The moderate agreement (K = 0.58) between ELISA and CLIA in IgM detection is due to the greater number of false positive results with ELISA. 

The excellent sensitivity of both assays in detecting IgM allows their use in the diagnosis of primary infection by *C. burnetii* since, in the early stages, IgM, together with PCR, are the first markers to be positive [21]. However, the low specificity of the ELISA IgM assay would require confirmation with another assay, such as the IFA assay. The CLIA IgM assay also has the advantage of greater specificity compared to the ELISA assay.

Cross-reactions between Coxiella, Legionella, and Bartonella species have been reported by IFA [14,15], and with *M. pneumoniae* and *B. pertussis* infections by ELISA [22]. In our study, we have not used sera from patients with other recently documented infections, although it would be interesting to investigate this cross-reactivity in these assays.

## 5. Conclusions

Overall, the results suggest that the CLIA IgM technique demonstrated generally superior performance compared to ELISA IgM, with better specificity, similar sensitivity, and a higher Kappa index. The sensitivity and specificity of the IgG CLIA assay and IgG ELISA are good, particularly that of the IgG ELISA assay, making both kits suitable for detecting this marker in the laboratory.

## Figures and Tables

**Table 1:** Comparison of CLIA and ELISA IgG results.

Classification	Positive ELISA	Negative ELISA	Equivocal ELISA	Overall
CLIA positive	69	5	3	77
CLIA negative	12	52	2	66
CLIA equivocal	4	1		5
Overall	85	58	5	148

**Table 2:** Comparison of CLIA and ELISA IgM results.

Classification	Positive ELISA	Negative ELISA	Equivocal ELISA	Overall
CLIA positive	26	4	0	30
CLIA negative	13	40	4	57
CLIA equivocal	1	0	0	1
Overall	40	44	4	88

**Table 3:** Comparison of CLIA and ELISA assays with indirect fluorescent antibody test (IFA).

		IFA Results (N)		Kappa Coefficient (95% CI)	Sensitivity, % (95% CI)	Specificity, % (95% CI)
		Positive	Negative			
Phase II Ig G						
CLIA	Positive	71	6	0.69 (0.57–0.80)	81 (71–88)	90 (80–95)
	Negative	17 ^a^	54			
ELISA	Positive	82	3	0.87 (0.80–0.95)	93 (88–98)	95 (89–100)
	Negative	6 ^b^	57			
Phase II IgM						
CLIA	Positive	24	7 ^c^	0.77 (0.56–0.97)	92 (82–100)	89 (81–97)
	Negative	2	55			
ELISA	Positive	25	19 ^d^	0.54 (0.35–0.73)	96 (89–100)	69 (58–80)
	Negative	1	43			

CI, confidence interval; ^a^ includes five equivocal results; ^b^ includes five equivocal; ^c^ includes one equivocal result; ^d^ includes four equivocal results.

## Data Availability

The raw data supporting the conclusions of this article will be made available by the authors on request.
